# Direct Reprogramming of Spiral Ganglion Non-neuronal Cells into Neurons: Toward Ameliorating Sensorineural Hearing Loss by Gene Therapy

**DOI:** 10.3389/fcell.2018.00016

**Published:** 2018-02-14

**Authors:** Teppei Noda, Steven J. Meas, Jumpei Nogami, Yutaka Amemiya, Ryutaro Uchi, Yasuyuki Ohkawa, Koji Nishimura, Alain Dabdoub

**Affiliations:** ^1^Biological Sciences, Sunnybrook Research Institute, Toronto, ON, Canada; ^2^Department of Otolaryngology – Head and Neck Surgery, Kyushu University, Fukuoka, Japan; ^3^Department of Laboratory Medicine and Pathobiology, University of Toronto, Toronto, ON, Canada; ^4^Division of Transcriptomics, Medical Institute of Bioregulation, Kyushu University, Fukuoka, Japan; ^5^Hearing Communication Medical Center, Shiga Medical Center Research Institute, Moriyama, Japan; ^6^Department of Otolaryngology – Head & Neck Surgery, University of Toronto, Toronto, ON, Canada

**Keywords:** cochlea, degenerative disease, regenerative medicine, reprogramming, neuron

## Abstract

Primary auditory neurons (PANs) play a critical role in hearing by transmitting sound information from the inner ear to the brain. Their progressive degeneration is associated with excessive noise, disease and aging. The loss of PANs leads to permanent hearing impairment since they are incapable of regenerating. Spiral ganglion non-neuronal cells (SGNNCs), comprised mainly of glia, are resident within the modiolus and continue to survive after PAN loss. These attributes make SGNNCs an excellent target for replacing damaged PANs through cellular reprogramming. We used the neurogenic pioneer transcription factor Ascl1 and the auditory neuron differentiation factor NeuroD1 to reprogram SGNNCs into induced neurons (iNs). The overexpression of both *Ascl1* and *NeuroD1 in vitro* generated iNs at high efficiency. Transcriptome analyses revealed that iNs displayed a transcriptome profile resembling that of endogenous PANs, including expression of several key markers of neuronal identity: Tubb3, Map2, Prph, Snap25, and Prox1. Pathway analyses indicated that essential pathways in neuronal growth and maturation were activated in cells upon neuronal induction. Furthermore, iNs extended projections toward cochlear hair cells and cochlear nucleus neurons when cultured with each respective tissue. Taken together, our study demonstrates that PAN-like neurons can be generated from endogenous SGNNCs. This work suggests that gene therapy can be a viable strategy to treat sensorineural hearing loss caused by degeneration of PANs.

## Introduction

Primary auditory neurons (PANs), also known as spiral ganglion neurons, transmit electrical signals from the inner ear to the central cochlear nucleus in the brainstem (Dabdoub et al., 2015). Once lost, PANs will not regenerate, resulting in permanent hearing impairment (White et al., 2000; Kujawa and Liberman, 2009). PAN degeneration is correlated with elevated pure tone audiometric thresholds and decreased word recognition scores, becoming progressively worse as more PANs are damaged (Sagers et al., 2017). Hearing aids and cochlear implants are auditory prosthetics that require the stimulation of PANs, either indirectly or directly, to transmit sound information to the brain, therefore patients lacking a suitable number of healthy PANs cannot benefit from these devices. However, if PANs could be replaced or regenerated, it might be possible to restore the hearing of patients with severely damaged PANs (Dabdoub and Nishimura, 2017). Additionally, regeneration of PANs may benefit individuals with hidden hearing loss, a condition correlated with PAN degeneration. Hidden hearing loss describes when individuals experience difficulty hearing in noisy settings but do not clinically present with auditory disability (see review Liberman and Kujawa, 2017).

One potential approach to treat hearing loss is the use of gene therapy. Cochlear gene therapy studies have typically focused on the preservation of remaining PANs through stimulation by neurotrophic factors such as brain-derived neurotrophic factor (BDNF) (Shibata et al., 2010; Wise et al., 2011), neurotrophin-3 (NT-3) (Wise et al., 2011; Suzuki et al., 2016), or human-nerve growth factor (Wu et al., 2011). These studies examined whether gene therapy could be used to preserve existing PANs and support the growth of neurites to cochlear hair cells, but did not try to replace lost neurons. Thus, far, cell transplantation has been the only strategy reported to introduce new neurons that facilitated partial recovery of hearing (Chen et al., 2012). However, transplantation remains a challenging task due to the anatomical barriers present within the cochlea. Transplanted cells are required to accurately migrate into the auditory system and, in the case of progenitor cells, are required to also cease proliferation to avoid tumor formation (Nishimura et al., 2012).

Cellular reprogramming is an alternative strategy that can be used to restore PANs and is an emerging area in the field of regenerative medicine (see review, Srivastava and DeWitt, 2016). We have previously reprogrammed cochlear non-sensory epithelial cells into induced neurons through the overexpression of the pioneer neurogenic transcription factor *Ascl1* (Nishimura et al., 2014). In the current study, we use spiral ganglion non-neuronal cells (SGNNCs) for cellular reprograming and neuron induction. SGNNCs are an optimal cell type for reprogramming since they reside in Rosenthal's canal surrounding PANs. SGNNCs are composed primarily of Schwann cells (Nayagam et al., 2011) with smaller populations of other mesenchymal cells. Schwann cells in the peripheral auditory system support and nourish PANs, therefore Schwann cells will also be necessary for the survival and stimulation of reprogrammed neurons (Whitlon et al., 2009). This means that a portion of the local Schwann cell population will need to be retained to support the growth of other reprogrammed cells. Fortunately, Schwann cells are abundant, continue to survive and even proliferate after PAN degeneration so there is little concern that cellular reprogramming will deplete Schwann cell reserves (Lang et al., 2011).

Ascl1, a pioneer neurogenic transcription factor, can alone convert various cell types into neurons *in vitro* at high efficiency, even at postnatal stages (Chanda et al., 2014; Nishimura et al., 2014). Ascl1 induces both GABAergic and glutamatergic neurons when reprogramming cortical astrocytes *in vitro* (Heinrich et al., 2010; Masserdotti et al., 2015), and induces mainly glutamatergic neurons when reprogramming midbrain astrocytes or mouse embryonic fibroblasts (Chanda et al., 2014). When delivered *in vivo*, either as part of a transcription factor cocktail (Torper et al., 2015) or on its own (Liu et al., 2015), Ascl1 has been shown to reprogram endogenous mouse astrocytes into neurons. Since PANs are glutamatergic neurons that stimulate the cochlear nucleus neurons of the brainstem (Zhou et al., 2007; Yuan et al., 2014), glutamatergic neuronal induction is necessary to create *bona fide* reprogrammed PANs that can reconstruct the auditory pathway. We hypothesized that NeuroD1, which is necessary for PAN development (Ma et al., 1998; Liu et al., 2000; Kim et al., 2001; Bell et al., 2008; Evsen et al., 2013) and is sufficient to induce neurons from embryonic cochlear non-sensory epithelial cells (Puligilla et al., 2010), together with Ascl1, could induce glutamatergic PAN-like neurons from SGNNCs. Here, we generated induced neurons (iNs) utilizing a combination of the transcription factors Ascl1 and NeuroD1, and performed transcriptome analyses to compare iNs to endogenous PANs and SGNNCs.

## Materials and methods

### Animals

Tau-EGFP knock-in mice (Tucker et al., 2001) (Jackson Laboratories, STOCK *Mapt*^*tm*1(*EGFP*)*Klt*^/J; stock number, 004779), which disrupts expression of the Mapt gene by replacing it with the EGFP coding sequence results in EGFP expression in neurons, and CD-1 mice (Charles River) were used. To obtain heterozygous pups, Tau-EGFP homozygous male mice were crossed with CD-1 female mice (Charles River laboratory). We used heterozygous Tau-EGFP knock-in pups since these animals are phenotypically comparable to CD-1 mice whereas homozygous Tau-EGFP knock-in animals displayed impaired contextual and cued fear conditioning, and severe deficits in long-term potentiation (Ahmed et al., 2014). Care and euthanasia of the mice used in this study were approved by Sunnybrook Research Institute Animal Care Committee, in accordance with IACUC regulations.

### Harvesting postnatal spiral ganglia

To collect spiral ganglia from postnatal day 1 (P1) Tau-EGFP mice, we dissected out the temporal bone, removed the cartilaginous capsule and the membranous labyrinth of the cochlea (Oshima et al., 2007). Then we gently separated the spiral ganglion from the modiolus. We collected 20–24 spiral ganglia in 100 μL of HBSS without CaCl_2_ and MgCl_2_ with 10 mM HEPES, 1 mM EDTA, and 0.1% BSA in 1.5 mL Eppendorf® tubes on ice. We digested the tissue as described previously (Oshima et al., 2007). Briefly, the tissues were digested with 50 μL of 0.25% trypsin/EDTA at 37°C for 15 min followed by enzymatic inactivation by adding 50 μL of 20 mg/mL soybean trypsin inhibitor (Worthington, cat no. LS003571) and 2 mg/mL DNase I solution (Worthington, cat no. LS002139) in Dulbecco's Modified Eagle Medium/Nutrient Mixture Ham's F-12 with GlutaMAX™ supplement (DMEM/Ham's F12 with GlutaMAX™ supplement 1 ×; Gibco, cat-no. 10565-042). The cell suspension was passed through a 40-μm cell strainer (BD Labware), centrifuged at 300 g for 5 min and resuspended in 500 μl of HBSS without CaCl_2_ and MgCl_2_, with 10 mM HEPES, 1 mM EDTA, and 0.1% BSA.

### Fluorescence activated cell sorting

Cells were sorted in a flow cytometer (FACSVantage) equipped with a 100 μm nozzle, and operated using the FACSDiva software (BD Biosciences). Distinct populations of cells were isolated based on forward scattering, lateral scattering, and the intensity of EGFP or DsRed fluorescence. Sorted cells were collected into Eppendorf® low-bind tubes with 100 μl HBSS without CaCl_2_ and MgCl_2_, with 10 mM HEPES, 1 mM EDTA, and 0.1% BSA. The Tau-EGFP negative fraction consisting of SGNNCs was further cultured for transfection with neurogenic factors or an empty vector. After culturing for 6–10 days, we collected induced neurons from the Tau-EGFP and DsRed double positive fraction. We also collected the Tau-GFP negative DsRed positive fraction transfected with an empty vector as a negative control. Lastly, we collected Tau-EGFP positive endogenous PANs at P1 to profile these cells and compare them to iNs.

### Plasmid DNA

We used a bi-cistronic expression vector pIRES2 DsRed-Express2 (Clontech), which allowed the simultaneous expression of our protein of interest and DsRed-Express2. A *pCMV-X-DsRed2*, where X is *Ascl1* or *NeuroD1*, was constructed by inserting the coding DNA sequence of X into the multiple cloning site of the pIRES2 DsRed-Express2 plasmid. Henceforth, we will use the “X-DsRed” notation to refer to pCMV-X-DsRed2 vector.

### Transfection and cell culture

We cultured cells of the spiral ganglia from CD-1 mice or sorted PANs/SGNNCs from Tau-EGFP mice on glass bottom dishes coated with 0.2% gelatin in “astro-medium” containing DMEM/Ham's F12 with GlutaMAX™ supplement with 10% heat inactivated FBS (Invitrogen), 5% heat inactivated horse serum, 2% B27 supplement (Thermo Fischer Scientific), 10 ng/ml EGF (Sigma-Aldrich), 10 ng/ml bFGF (Invitrogen), and 6.0 mg/ml of D-(+)-glucose (Heinrich et al., 2010). At 60–70% confluency, we transfected cells with the expression vector(s). Cells were transfected using Lipofectamine LTX (Life Technologies) based on previously published methods (Heinrich et al., 2010) and manufacturer's instructions. We incubated the cells in DNA/Lipofectamine complexes for 4 h at 37°C with 5% CO_2_. After replacing DNA/Lipofectamine complexes with astro-medium and incubating for 20 h, the cells were maintained in “differentiation medium” consisting of DMEM/Ham's F12 with GlutaMAX™ supplement, 2% B27 supplement, 1% N2 supplement, 10 ng/ml of BDNF (Peprotech), 10 ng/ml of NT-3 (Peprotech) and 6.0 mg/ml of D-(+)-glucose (Oshima et al., 2007) for an additional 6 to 10 days.

### Co-culture of induced neurons with target tissue

For co-culture experiments, P1 SGNNCs were cultured and transfected with *Ascl1-DsRed*, and maintained for 3 days on glass bottom dishes coated with 0.2% gelatin in astro-medium. After we dissociated transfected cells with trypsin-EDTA (0.25%) (Gibco), cells were re-suspended in 500 μl of differentiation medium and co-cultured with embryonic day 13.5 (E13.5) CD-1 cochlear epithelium or P1 Tau-GFP cochlear nucleus on Matrigel-coated glass-bottom dishes. Cultures were maintained for an additional 7 days *in vitro*.

### Immunostaining

Induced Immunostaining was performed on cochlear cross sections and cells in culture. Cochleae or cells were washed with PBS and fixed with 4% paraformaldehyde for 30 min to 2 h at room temperature. After washing three times with PBS, cochleae were cryopreserved in OCT compound (Sakura), sectioned and mounted on glass slides (Fisher). Cochlear cross sections or fixed cells were permeabilized and blocked in a solution of PBS containing 10% donkey serum and 0.5% TritonX-100 (Sigma) for 30 min at room temperature. Samples were incubated in the primary antibodies diluted in PBS containing 10% donkey serum and 0.5% TritonX-100 (Sigma) overnight at 4°C. The following day, samples were washed three times with PBS and incubated in the secondary antibody solution for 1 h at room temperature. Samples were washed three times with PBS, mounted on glass slides and imaged using a confocal microscope SP5 (Leica). The following antibodies were used: mouse anti-MAP2 (Sigma, 1:300), rabbit anti-TuJ1 (for βIII-tubulin) (Sigma, 1:1000), rabbit anti-VGLUT1 (Synaptic systems, 1:5000), goat anti-hProx1 (R&D, 1:250), and goat anti-Sox10 (Santa Cruz, 1:200).

### Calculating neuronal conversion efficiency

Induced cells derived from P1 CD-1 mice were counted from a minimum of six different dishes per condition and at least three independent experiments (different litters). We followed a previously reported protocol for evaluating neuron induction (Pang et al., 2011). In order to keep counts unbiased, transfected cells were selected at random based on the expression of the DsRed marker. These cells were assayed for the expression of neuronal markers βIII-tubulin (TuJ1) (Caccamo et al., 1989) or microtubule-associated protein 2 (MAP2) (Hafidi et al., 1992). Within the group of TuJ1- or MAP2-positive cells, induced neurons were quantified based on the criteria that they had at least one neurite extension that was three times longer than its cell body. The average value for each litter was used in the analysis. All data were presented as the mean ± standard error of the mean (SEM). Differences in neuron induction efficiency among different combinations of transcription factors were examined using two-factor ANOVA, followed by Tukey-Kramer test for multiple comparisons. *p* < 0.05 were considered to be significant.

### Transcriptome analysis (RNA-seq)

RNA was extracted using the Single Cell RNA Purification Kit (NORGEN, #51800) from each of the following groups; Tau-EGFP positive endogenous PANs, DsRed (Ascl1 and NeuroD1) and Tau-EGFP positive iN, and DsRed positive vector-control (VC). The quality of extracted RNA was verified by Bioanalyzer 2100 RNA 6000 pico chip (Agilent Technologies) and the concentration was measured by Qubit RNA HS Assay (Thermo Fisher). RNA library preparation was performed using a two-pronged approach: (1) Two ng of input RNA was converted to double stranded cDNA using Clontech SMARTer Ultra Low Input RNA Kit v3 using Clontech's proprietary Switching Mechanism at 5' End of RNA Template (SMART) technology, following the manufacturer's instructions; double stranded (ds) DNA was then quantified by Qubit HS assay and then (2) 1 ng of ds-DNA was used as input material for the Nextera XT library preparation following Illumina's recommended protocol. One microliter of the final RNA-Seq libraries was loaded on a Bioanalyzer 2100 DNA High Sensitivity chip to check for size; RNA libraries were quantified by qPCR using the Kapa Library Quantification Illumina/ABI Prism Kit protocol (KAPA Biosystems). Libraries were pooled in equimolar quantities and paired-end sequenced on an Illumina HiSeq 2500 platform using a High Throughput Run Mode flowcell and the V4 sequencing chemistry following Illumina's recommended protocol to generate paired-end reads of 126-bases in length. Three biological repeats of each group were loaded and sequenced at 40M reads. We mapped FASTQ files to mm10 genome by HISAT2 protocol (Kim et al., 2015; Pertea et al., 2016), which is a refined protocol based on TopHat2 (Kim et al., 2013). Expression data were normalized by the transcripts per million reads (TPM) method (Wagner et al., 2012), which enables us to avoid statistical biases inherent in the traditional read per kilobase per million reads (RPKM) method (Mortazavi et al., 2008). Genes were included in our analysis if at least one of the three samples surpassed a threshold of 0.2 TPM. Using this method, we detected a total of 17,601 genes. Raw RNA-seq data have been deposited in the NCBI Gene Expression Omnibus (GEO) under accession number GSE107461.

### Droplet digital PCR (ddPCR)

We used Droplet digital PCR (Hindson et al., 2011) for validation of RNA-seq data. The QX200 droplet digital PCR (ddPCR) system (Bio-Rad) was used to quantify the absolute number of βIII-tubulin, Prph, Sox2 and Tnc mRNA molecules in cells from each of the three groups. Extracted RNA was amplified and reverse transcribed to cDNA using CellAmp Whole Transcriptome Amplification Kit Ver.2 (Clontech). We utilized Taqman probes (Applied Biosystems) for βIII-tubulin (Mm00727586_s1), Prph (Mm00449704_m1), Sox2 (Mm03053810_s1), and Tnc (Mm01262852_m1). PCR was performed in 20 μl containing 0.52 ng of cDNA, 900 nM of the forward and reverse primers, 250 nM probe, and 10 μl of 2X ddPCR supermix for probes (Bio-Rad). Each ddPCR assay mixture was loaded into a disposable droplet generator cartridge (Bio-Rad). Then, 70 μL of droplet generation oil for probes (Bio-Rad) was loaded into each of the eight oil wells. The cartridge was then placed inside the QX200 droplet generator (Bio-Rad). When droplet generation was completed, the droplets were transferred to a 96-well PCR plate (Eppendorf) using a multichannel pipette. The plate was heat-sealed with foil and placed in a C1000 Touch Thermal Cycler (Bio-Rad). Thermal cycling conditions were as follows: 95°C for 10 min, followed by 44 cycles of 94°C for 30 s and 60°C for 1 min, then 98°C for 10 min, and a 4°C indefinite hold. FAM fluorescent signal in each droplet was counted by QX200 digital droplet reader and analyzed by QuantaSoft analysis software ver.1.7.4.0917 (Bio-Rad).

## Results

### Spiral ganglion non-neuronal cells can be reprogrammed into neurons

**Figure 1:**
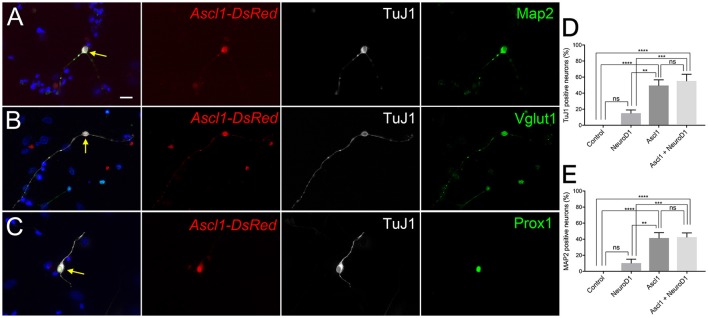
*Ascl1* can induce SGNNCs into cells with neuronal characteristics. *Ascl1* reprogrammed SGNNCs into induced neurons expressing PAN-specific neuronal markers. Dissociated SGNNCs (WT) were transfected with *Ascl1-DsRed* and cultured for 8 days *in vitro* (DIV), followed by immunocytochemistry with DAPI labeling cell nuclei (blue). **(A)** An induced neuron (indicated by arrow) transfected with *Ascl1-DsRed* (red) expressed pan-neural marker βIII-tubulin (labeled by TuJ1; white), and mature PAN marker MAP2 (green). **(B)** An induced neuron (indicated by arrow) transfected with *Ascl1-DsRed* (red) expressed TuJ1 (white), and a glutamatergic neuronal marker Vglut1 (green). **(C)** An induced neuron (indicated by arrow) transfected with *Ascl1-DsRed* (red) expressed TuJ1 (white), and a PAN-specific transcription factor Prox1 (green). **(D,E)** Comparison of neuronal induction efficiency using each or a combination of the transcription factors Ascl1 and NeuroD1. Neuronal marker TuJ1 **(D)** or MAP2 **(E)** positive cells were quantified in DsRed positive transfected cells. *n* = 6 for each experiment. Asterisks in **(D,E)** indicate significant difference (^**^p < 0.01, ^***^p < 0.001, ^****^p < 0.0001). Error bars indicate standard error of the mean (SEM). Scale bar in A, 20 μm.

We wanted to examine the potential for *Ascl1* to reprogram SGNNCs into induced neurons. Six to ten days post-transfection, 49 and 41% of wild-type SGNNCs transfected with *Ascl1* expressed common neuronal markers TuJ1 and MAP2, respectively, and exhibited a neuronal morphology (Figures 1A,D). iNs also expressed vesicular glutamate transporter 1 (VGLUT1) (Figure 1B), which packages glutamate into synaptic vesicles (Zhou et al., 2007; Yuan et al., 2014), and the transcription factor Prox1 (Figure 1C), which is expressed in developing PANs, mature type I PANs and the support cells of the organ of Corti (Bermingham-McDonogh et al., 2006; Karalay, 2011; Nishimura et al., 2017). These results indicate that *Ascl1* has the ability to induce neuron-like cells from SGNNCs.

Although Ascl1 is a robust pioneer and neurogenic factor, it is not known whether it plays a role in normal PAN development. Since NeuroD1 is known to be a necessary factor in PAN development and differentiation, we chose to ectopically express NeuroD1 in addition to Ascl1 (Liu et al., 2000; Kim et al., 2001; Evsen et al., 2013). NeuroD1 can alone induce glutamatergic neurons from cortical glial cells (Guo et al., 2014). Further, the combination of NeuroD1 and Ascl1 can induce neuron-like cells from organ of Corti derived non-sensory epithelial cells that are more electrophysiologically similar to endogenous neurons than Ascl1 alone (Nishimura et al., 2014). Thus, we hypothesized that overexpression of *NeuroD1* in addition to *Ascl1* could induce neurons that are closer to PANs than *Ascl1*-only induced neurons. We co-transfected SGNNCs with *Ascl1* and *NeuroD1*, and observed that co-expression induced neurons at a similar efficiency (Figure 1D, E, 55 vs. 49%). Based on a two-factor ANOVA, *Ascl1* transfection was responsible for the main effect in conversion efficiency [Ascl1: *F*_(1, 20)_ = 55.02, *p* < 0.0001; NeuroD1: *F*_(1, 20)_ = 1.309, *p* = 0.2661] and there were no interacting effects between *Ascl1* and *NeuroD1* [*F*_(1, 20)_ = 0.8289, *p* = 0.3734]. Multiple comparisons performed using the Tukey-Kramer method demonstrated that both *Ascl1* alone, and *Ascl1* and *NeuroD1* co-transfection induced neurons at significantly higher efficiency than empty vector (*p* < 0.0001; *p* < 0.0001) or *NeuroD1* alone (*p* < 0.002; *p* < 0.001). There were also no significant differences between *Ascl1* alone and *Ascl1 and NeuroD1* co-transfection (*p* = 0.9983). However, we continued using the combination of Ascl1 and NeuroD1 based on our previous results indicating co-transfected cells induced neurons that were more mature.

### Comparing the transcriptome of iNs, PANs, and VCs using RNA-seq and digital droplet PCR

**Figure 2:**
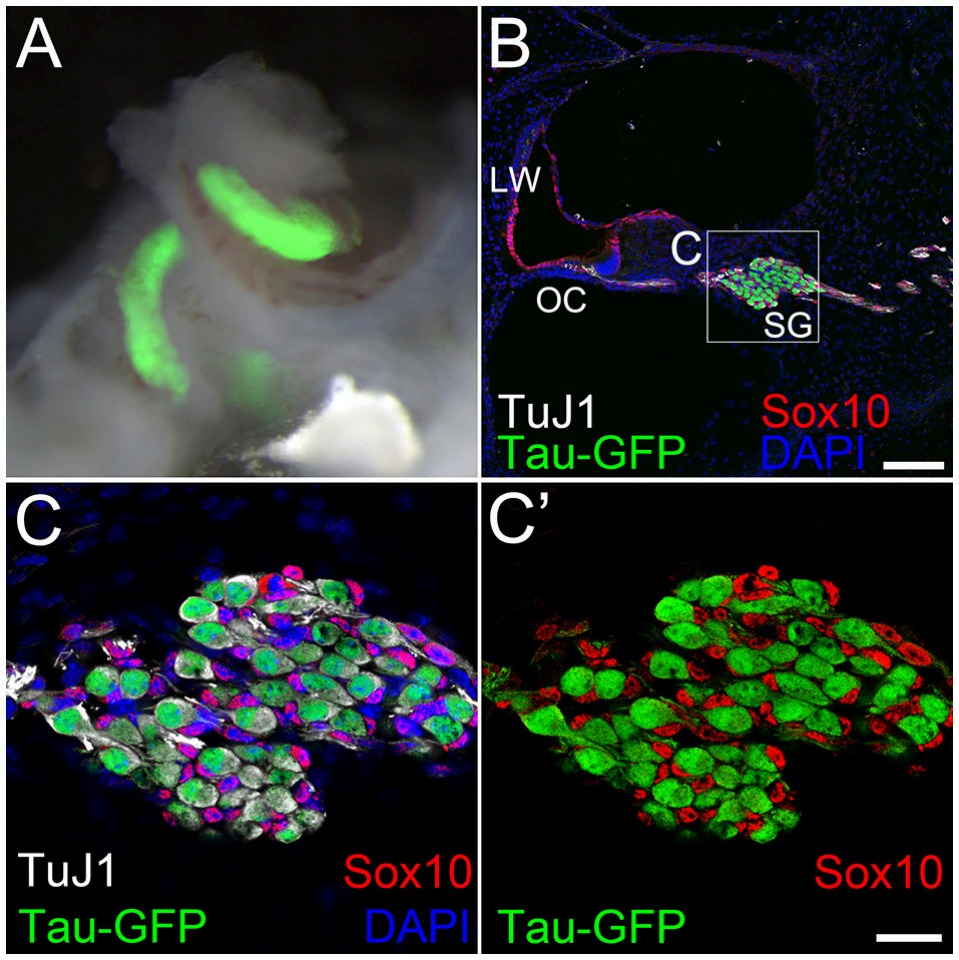
Tau-EGFP marks primary auditory neurons. **(A)** A bright field image of P1 Tau-EGFP spiral ganglion overlapped with Tau-EGFP fluorescence (green). **(B)** Low magnification view of a cochlear cross section of P1 Tau-EGFP mouse reporter showing Tau-EGFP (green) and TuJ1 (white) positive primary auditory neurons. Glial cells in the spiral ganglion and cells in the cochlear duct expressed Sox10 (red). All cell nuclei were labeled with DAPI (blue). **(C)** High magnification view of spiral ganglion in **(B)** (dotted box). **(C**′**)** The same image as in **(C)** without DAPI channel. LW; lateral wall, OC; organ of Corti, SG; spiral ganglion. Scale bar in **(B)** 100 μm. Scale bar in **(C**′**)**, 20 μm.

**Figure 3:**
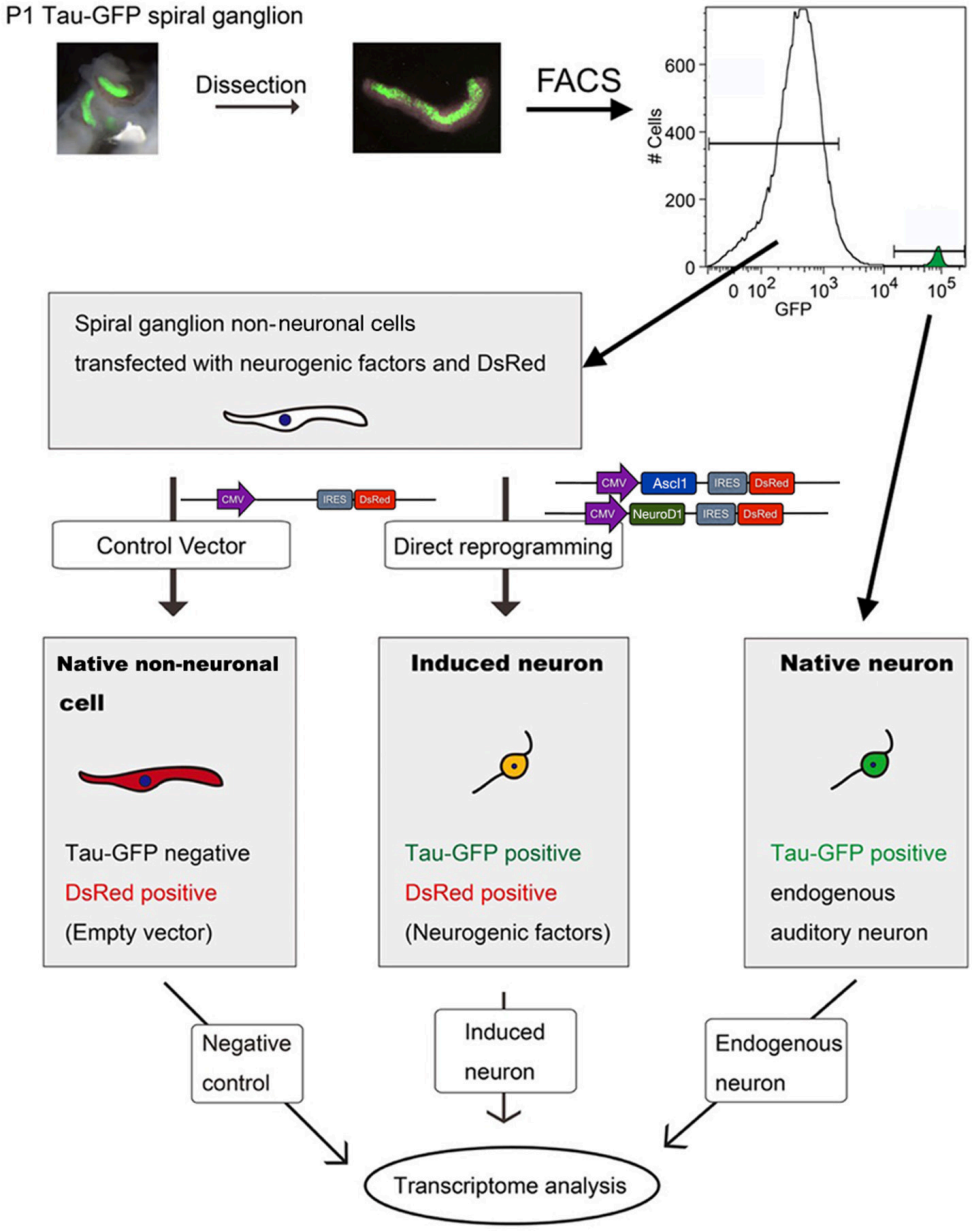
Work flow for transcriptome analyses. P1 Tau-EGFP cochleae were dissected and isolated SGs were sorted by EGFP fluorescence with flow cytometer FACSVantage. EGFP positive fraction contained endogenous PANs, and EGFP negative fraction containing SGNNCs. SGNNCs were cultured and transfected with transcription factors or control vector -induced neurons (iN) or vector control (VC) respectively. PANs, iNs, and VC cells were collected and RNA extracted for transcriptome analysis.

**Figure 4:**
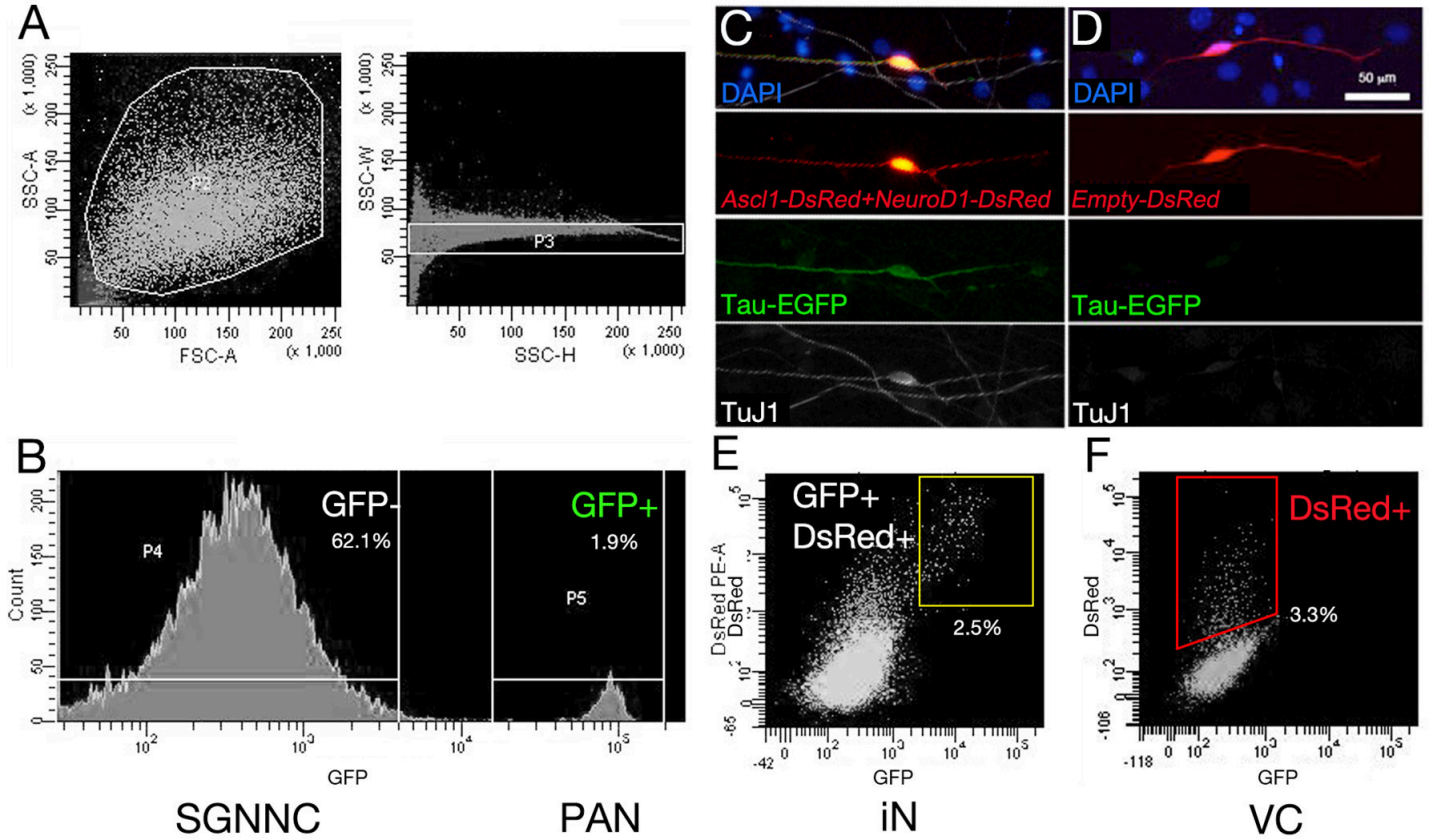
Sorting spiral ganglion cells and RNA collection. The procedure for RNA collection is indicated. **(A)** Tau-EGFP SG cells were sorted with a flow cytometer. Dead cells were excluded first, which have small side scatter (SSC) and forward scatter (FSC) parameters (Left panel in **A)**. Doublets were removed (Right panel in **A)**. **(B)** EGFP positive fraction was collected as endogenous PANs, and EGFP negative fraction was collected as SGNNCs. The latter was cultured and transfected with transcription factors or control vector. **(C)** Tau-EGFP was upregulated in Ascl1-DsRed and NeuroD1-DsRed double transfected cells, which also expressed neuronal marker TuJ1. **(D)** Empty-DsRed-transfected cells expressed only DsRed, however GFP was not upregulated and TuJ1 was not expressed. **(E)** EGFP and DsRed double positive cells were collected as iNs from *Ascl1*- and *NeuroD1*-transfected cells. **(F)** DsRed single positive cells were collected as vector-control (VC) from *Empty-DsRed*-transfected cells. Scale bar: 50 μm.

To ensure that our starting cell population was not contaminated with endogenous neurons we performed fluorescence-activated cell sorting using EGFP expression to separate the neuronal and non-neuronal populations. We used *Tau*^*EGFP*^^/+^ (Tau-EGFP) mouse cochlea for these experiments, where EGFP is expressed only in neurons. Gross morphology of P1 mouse cochlea after removal of the sensory epithelium indicated that PANs were EGFP positive (Figure 2A). Cochlear cross sections from Tau-EGFP mice demonstrated that PANs co-expressed EGFP and βIII-tubulin, validating the use of EGFP as a neuronal marker in these cells (Figures 2B,C). Spiral ganglion glial cells surrounding PANs identified using Sox10 did not express EGFP (Figures 2B,C′). To purify Tau-EGFP^+^ PANs, we collected spiral ganglia from *Tau*^*EGFP*^^/+^ mice at P1 (refer to experimental design in Figure 3). From one litter of heterozygous pups, we collected 20–24 spiral ganglia that were subsequently dissociated and sorted based on EGFP fluorescence. After excluding dead cells and debris (Figure 4A), we regularly obtained 20,000–24,000 Tau-EGFP positive cells from one sort (~3% of total sorted cells; Figure 4B). Approximately, 900 endogenous PANs were isolated from one spiral ganglion (897 ± 174 Tau-EGFP^+^ cells and 26,953 ± 8,467 Tau-EGFP^−^ cells per spiral ganglion; *n* = 6). This number of sorted neurons is consistent with previous results using *Mafb-GFP* mice (Lu et al., 2011). We also collected the Tau-EGFP negative fraction, which included glial cells and non-neuronal, non-glial cells. Tau-EGFP negative cells were cultured for 2–3 days until 70% confluent and then were co-transfected with equal concentrations of *Ascl1*- and *NeuroD1-DsRed* or a *DsRed* control vector. After culturing for 6 to 10 days *in vitro*, cells transfected with *Ascl1* and *NeuroD1* displayed a neuron-like morphology, and expressed Tau-EGFP and βIII-tubulin (Figure 4C), whereas cells transfected with the control vector expressed only *DsRed* (Figure 4D). Using time-lapse live imaging we observed the real-time reprogramming of an SGNNC into a cell morphologically resembling a neuron (see Supplemental Video S1). To obtain a purified population of iNs and vector control (VC) cells for further analyses we collected these cells and once more sorted them based on EGFP and DsRed fluorescence. The EGFP- and DsRed-positive fraction, representing iNs, from *Ascl1*-*DsRed* and *NeuroD1*-*DsRed*-transfected cells were collected (Figure 4E). The EGFP-negative, DsRed-positive fraction, representing VCs, from *Empty-DsRed*-transfected cells were collected (Figure 4F). We extracted RNA from 3 biological replicates for each population: 1. endogenous PANs; 2. induced neurons (iNs); and 3. vector control cells (VCs).

**Figure 5:**
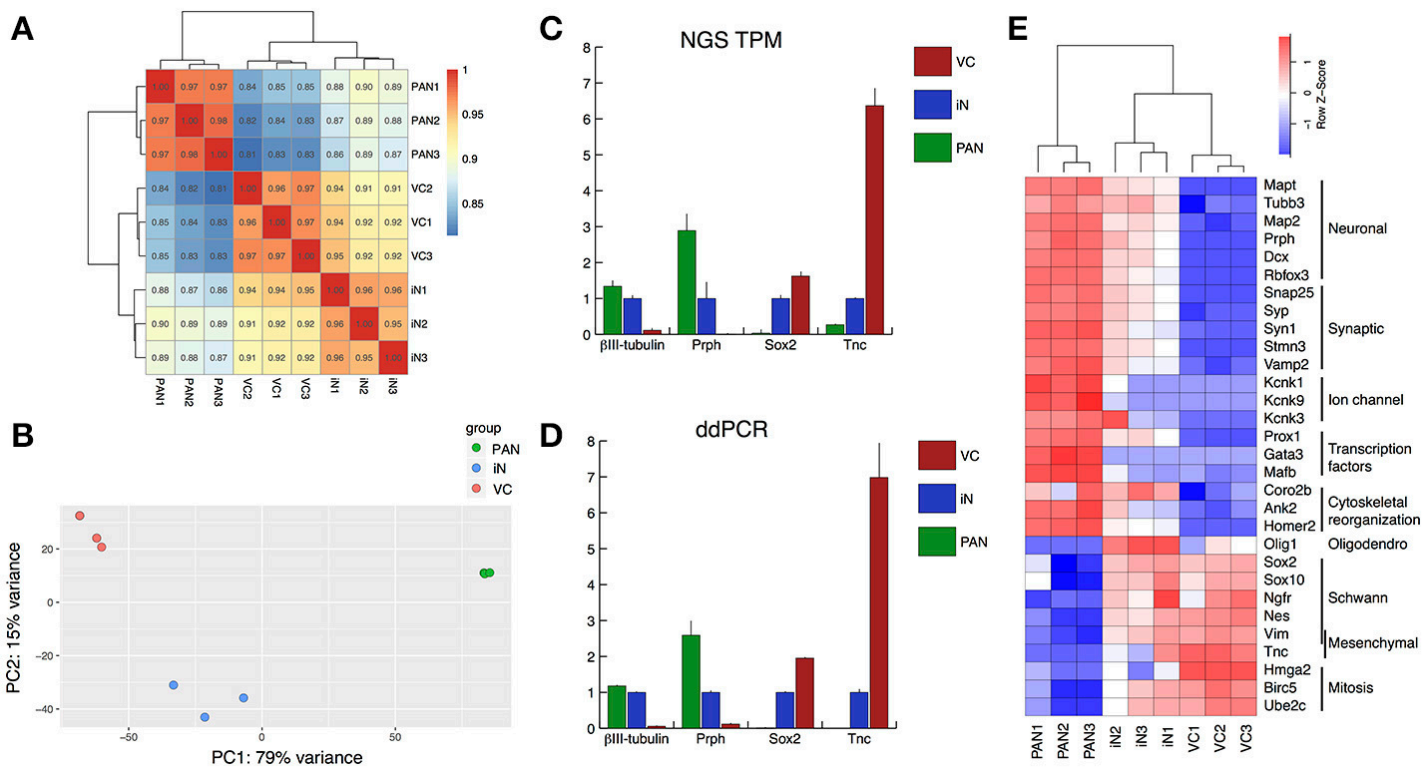
Validation of RNA-seq by droplet digital PCR, and gene expression profiling of neuron-/Schwann cell-/fibroblast-specific genes. **(A)** Heatmap showing Pearson correlations among samples in terms of the common logarithmic counts of genes with non-zero counts. Samples in each group correlated well with each other and the largest difference was between PAN and VC. **(B)** Principal component analysis (PCA). The regularized logarithmic transformation was applied to the normalized counts and PCA was performed on the top 500 most variable genes across samples. Three samples in each group were well clustered. **(C,D)** Validation of RNA-seq data using droplet digital PCR (ddPCR). Relative value of each group compared to iN (= 1.00) is shown. **(C)** Relative value of TPM was calculated for known neuron markers (TuJ1 and Prph), glial marker (Sox2), and fibroblast marker (Tnc). **(D)** Relative value of ddPCR expression data for the same 4 genes validated our RNA-seq data. Error bars represent standard error of the mean (SEM). **(E)** A heatmap for reportedly specific genes for pan-neuronal, synaptic, ion-channel, transcription factors, cytoskeletal reorganization, oligodendrocytes, Schwann cells, mesenchymal cells and mitosis. Red-white-blue spectrum indicates high-moderate-low expression level for each gene.

To investigate gene expression patterns among the endogenous PANs, iNs and VCs, we performed transcriptome analysis using next generation sequencing (Mortazavi et al., 2008). The sequenced data were mapped using the HISAT2 program (Kim et al., 2015; Pertea et al., 2016). The three groups (PAN: endogenous PANs; iN: induced neurons; and VC: vector controls) aggregated together after unbiased hierarchical clustering based on Pearson correlation values between samples (Figure 5A, Supplemental Figure S1). These three groups also emerged as separate clusters on a 2-dimensional PCA plot (Jolliffe, 1988; Love et al., 2014) encompassing 94% of the 500 genes with the largest variance (Figure 5B). PCA1 (x-axis) was responsible for 79% of the observed variance (Top 5 genes and weights; *Clrn1* 0.072, *Cpne4* 0.066, *Slc17a6* 0.064, *Nrg3* 0.064, *Ppp1r1c* 0.063). iNs were observed to form a noticeable cluster between the PAN and VC clusters, suggesting that iNs partly acquired neuronal characteristics. To validate our RNA-seq data we compared the expression of four genes with RNA-seq and droplet digital PCR (ddPCR) (Hindson et al., 2011): *Tubb3* (βIII-tubulin) and *Prph* (Caccamo et al., 1989; Hallworth and Ludueña, 2000; Barclay et al., 2011; Nishimura et al., 2017), *Sox2* (Lang et al., 2011; Nishimura et al., 2017), and *Tnc* (Chiquet Ehrismann and Chiquet, 2003) were chosen as neuronal, glial, and mesenchymal cell markers, respectively. The expression levels of these four genes were highly comparable between RNA-seq and ddPCR (Figures 5C,D), indicating that transcripts per million reads (TPM) from the RNA-seq data reflected the absolute value of each gene expression. Based on these results, we next profiled gene expression of PANs and iNs using a modified gene list described previously (Treutlein et al., 2016) (Figure 5E). *Mapt* (Tau) was expressed highly in PANs and moderately in iNs. Both PANs and iNs expressed neuronal markers *Tubb3, MAP2, Prph, Dcx, Rbfox3* (Kim et al., 2009); genes for synaptic proteins *Snap25* (Flores-Otero and Davis, 2011; Wang et al., 2013), *Stmn3, Vamp2, Syp* (Khalifa et al., 2003), *Syn1* (Scarfone et al., 1991); the gene for the ion channel *Kcnk3* (Chen and Davis, 2006) and the transcription factor *Prox1* (Bermingham-McDonogh et al., 2006) (Nishimura et al., 2017). However, the expression of genes for ion channels *Kcnk1* and *Kcnk9* (Chen and Davis, 2006), and transcription factors *Gata3* (Appler et al., 2013; Nishimura et al., 2017) and *Mafb* (Yu et al., 2013; Nishimura et al., 2017) were only expressed in endogenous PANs. iNs demonstrated lower expression levels of the glial marker *Nes* (Lang et al., 2015) and mesenchymal cell marker *Tnc* as compared to VCs. However, iNs still expressed genetic markers of VCs such as transcription factors *Sox2* and *Sox10*, markers of cochlear glial cells (Breuskin et al., 2010), *Ngfr*, a marker of glial cells (Provenzano et al., 2011), and *Vim*, a marker of mesenchymal tissue (Whitlon et al., 2010). *Olig1*, which encodes a transcription factor that promotes oligodendrocytes differentiation (Zhou and Anderson, 2002), was highly expressed only in iNs. At the same time, the expression of genes for cytoskeletal reorganization such as *Coro2b, Ank2*, and *Homer2* were increased in iNs, similar to PANs. The expression of mitotic genes such as *Hmga2, Birc5, Ube2c* were also decreased in iNs. Overall, our transcriptome analysis revealed that co-expression of *Ascl1* and *NeuroD1* could reprogram cells into induced neurons.

### Transcriptional profiling of iNs and PANs

**Figure 6:**
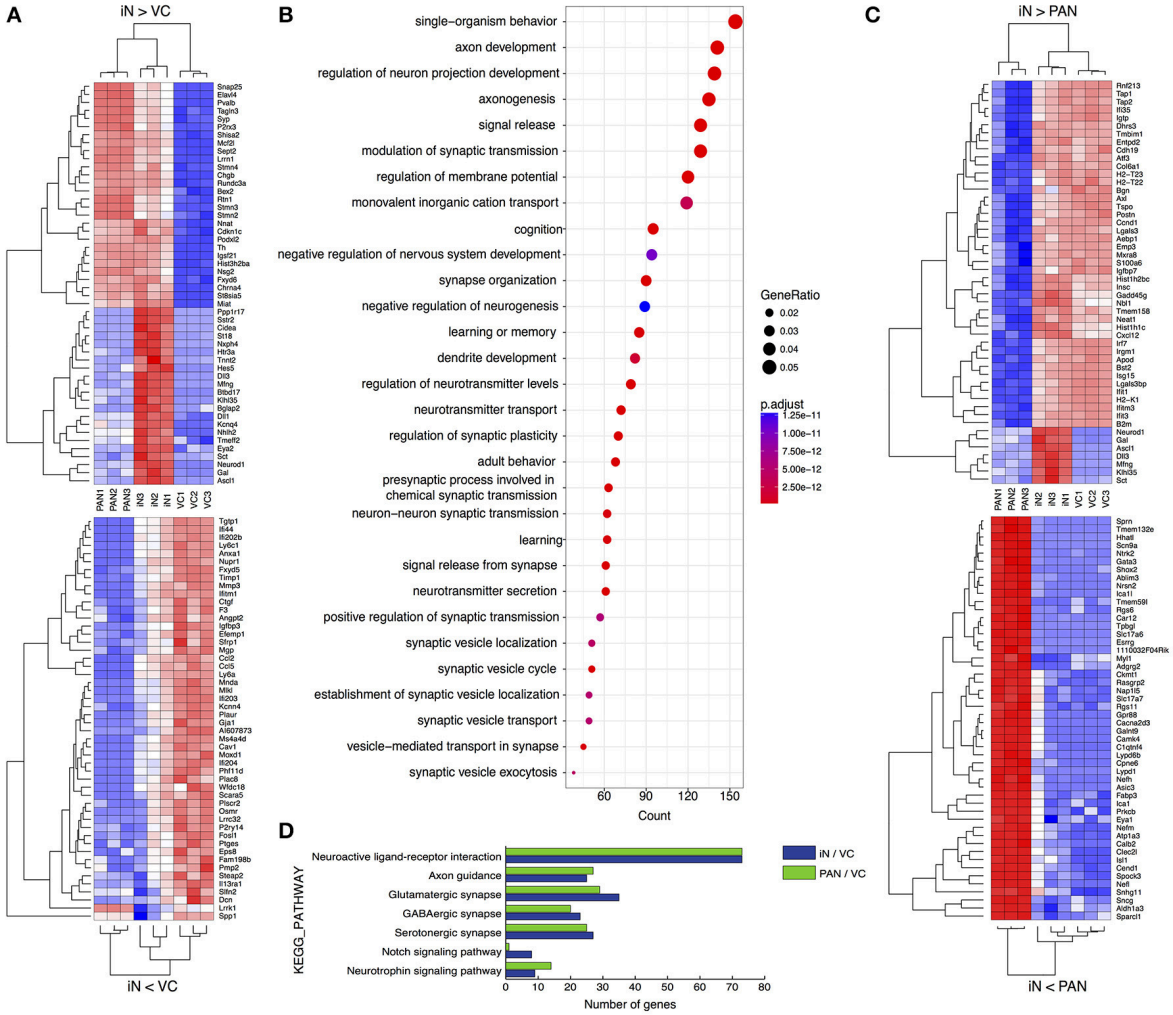
Comparative transcriptome analyses of PANs, iNs, and VCs. **(A)** Heatmaps drawn for 50 positively (upper) or negatively (lower) variant genes between iN and VC. **(B)** Gene ontology analysis for neuronal induction. Upregulated DEGs were extracted in iN against VC and performed GO analysis. The top 30 biological process terms are shown. The diameter of each circle indicates the gene ratio. The color of each circle indicates adjusted p value as shown to the right of the panel. **(C)** Heatmaps drawn for 50 positively (upper) or negatively (lower) variant genes between iN and PAN. **(D)** Pathway analysis comparing PAN and iN. Upregulated DEGs of PAN and iN against VC were analyzed on KEGG database.

We next profiled the transcriptome of iNs and PANs to analyze and compare the characteristics of each. We performed hierarchical clustering on genes with the 50 most positive and 50 most negative fold changes between iNs and either VCs or PANs. When we compared iNs and VCs (Figure 6A-upper), genes expressed higher in iNs were subdivided into 2 clusters: iN-high, PAN-high, VC-low and iN-high, PAN-low, VC-low. The former contains the PAN marker gene *Pvalb* (Kim et al., 2016) and genes for synaptic proteins *Snap25, Stmn2*/*3*, and *Syp*, demonstrating that iNs acquired PAN characteristics. Transcription factors *Ascl1* and *NeuroD1* were expressed here as expected. Of note, only iNs expressed factors that are involved in Notch signaling such as *Dll1, Dll3, Hes5*, and *Mfng* (Kopan and Ilagan, 2009). In contrast, we found the Schwann cell marker gene *Mgp* (Schmid et al., 2014) and the fibroblast specific gene *Dcn* (Danielson et al., 1997) in the iN-low, VC-high cluster, demonstrating iNs lost characteristics of SGNNCs (Figure 6A-lower). To identify which pathways were affected during iN induction from SGNNCs, we next extracted 4,091 differential expressed genes (DEGs) between the iN and VC groups and performed gene ontology analysis (Ashburner et al., 2000). Thirty neurogenic events were selected by gene ontology (Figure 6B). Genes that play a role in neuronal development such as axon development, regulation of neuron projection development, and axonogenesis were highly weighted in the top 30 biological processes. When we compared gene expression levels between iNs and PANs, iN-highly-expressed genes contained histone protein coding genes; immunological genes such as *Ifit1, Ifit3, H2-T22*, and *T23* (Ohtsuka et al., 2008; Abbas et al., 2013); as well as *Ascl1* and *NeuroD1* (Figure 6C-upper). In contrast, genes expressed at a lower level in iNs included genes that play a role in PAN development such as *Gata3* (Appler et al., 2013; Nishimura et al., 2017), *Eya1* (Ahmed et al., 2012), and *Isl1* (Radde-Gallwitz et al., 2004), suggesting iNs lacked some genes expressed in nascent PANs at least at this time point of analysis (Figure 6C-lower).

To compare the neuronal characteristics of iNs with PANs, we applied 2000 DEGs between either iNs or PANs and VCs using the KEGG PATHWAY database (Kanehisa and Goto, 2000) on the Database for Annotation, Visualization and Integrated Discovery (DAVID) (Huang et al., 2009). Pathway analysis revealed genes that play a role in synaptic development and maturation (e.g., “neuroactive ligand-receptor interaction” pathway, “glutamatergic synapse,” “axon guidance” etc.) were enriched both in PANs and iNs (Figure 6D). However, genes involved in neurotrophin signaling pathways were more enriched in PANs than iNs. Additionally, genes involved in Notch signaling were upregulated in iNs, which might have been a consequence of Ascl1 overexpression. Regardless, iNs expressed genes involved in pathways typically associated with PANs and not VCs.

### iNs extend projections both cochlear hair cells and cochlear nucleus neurons *in vitro*

**Figure 7:**
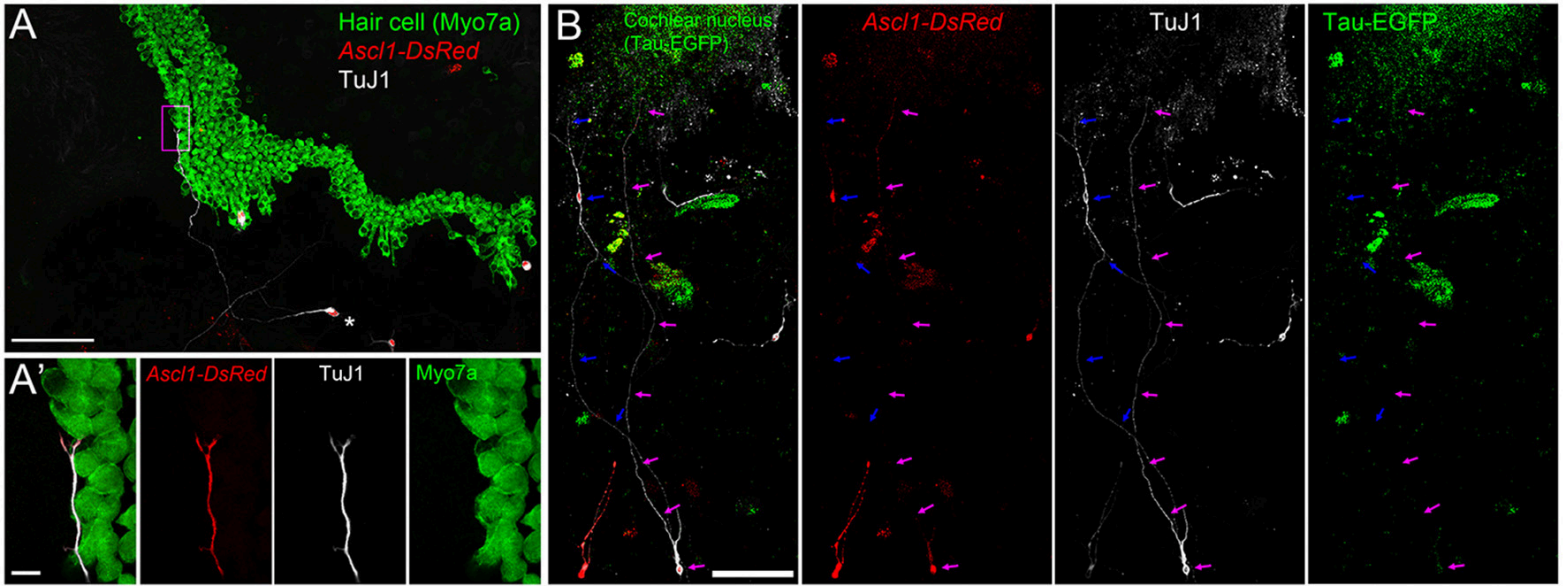
iNs extend projections toward cochlear hair cells and cochlear nucleus neurons. **(A)** Low-magnification image of CD-1 P1 SGNNCs transfected with *Ascl1-DsRed* (red) and co-cultured with a denervated CD1 E13.5 cochlear epithelium and maintained for 7 days *in vitro*, immunostained for TuJ1 (white; neuron marker) and Myo7a (green; sensory hair cell marker). **(A**′**)** High magnification image of box in **(A)**. Merged views are shown on the left and individual channels are to the right. ^*^Indicates cell body of iN. **(B)** Low-magnification image of CD-1 P1 SGNNCs transfected with *Ascl1-DsRed* (red) and co-cultured with a P1 Tau-EGFP cochlear nucleus (green) and maintained for 7 days *in vitro*, immunostained against the neuronal marker TuJ1 (white). Merged views are shown on the left and individual channels are to the right. Arrows in different colors indicate neuron extensions from individual induced neurons. Scale bar in **(A,B)**, 100 μm. Scale bar in A′, 10 μm.

To evaluate whether iNs can reach PAN targets, we co-cultured iNs with organ of Corti (OC) or cochlear nucleus (CN) explants. We cultured iNs, from wild type P0 SGNNCs transfected with *Ascl1-DsRed* alongside E13.5 cochlear explants (Figure 7A) and observed neurite extension from TuJ1-positive iNs to Myo7a-positive hair cells (Figure 7A′). In addition, we could identify neurite extensions from iNs to Tau-EGFP positive CN explants of P0 mice (Figure 7B). These data suggest that iNs are able to grow neurites toward targets from the peripheral auditory system and the central nervous system *in vitro*.

## Discussion

This was the first proof-of-principle study demonstrating that neuron-like cells could be induced from cells that naturally exist in the spiral ganglion along with PANs. We predicted that SGNNCs were likely to develop into glutamatergic PAN-like neurons through ectopic overexpression of the neurogenic transcription factors *Ascl1* and *NeuroD1*. We supplemented *Ascl1*, which can alone convert various cell types (Chanda et al., 2014; Treutlein et al., 2016), including cochlear non-sensory epithelial cells, into functional iNs (Nishimura et al., 2014) with *NeuroD1*. NeuroD1 was chosen since it is one of the primary factors which specifies neuronal fate and differentiation during PAN development (Ma et al., 1998; Liu et al., 2000; Kim et al., 2001; Bell et al., 2008; Evsen et al., 2013). We have previously shown that NeuroD1 also promotes more mature iNs in cochlear non-sensory epithelial cells (Nishimura et al., 2014). Therefore, the combination of *Ascl1* and *NeuroD1* achieved our aim of coaxing cells into becoming more PAN-like. We also examined whether we could induce neurons with *NeuroD1* on its own, but overexpression of *NeuroD1* alone could only convert SGNNCs into neuron-like cells at a low efficiency (Figures 1D,E). It may be possible that NeuroD1 is a less potent reprogramming factor or that it acts only on certain cell types such as auditory stem cells (Diensthuber et al., 2014) or reactive glia, whereas Ascl1 is a more broad actor that induces neurons from many different cell types (Wapinski et al., 2013; Chanda et al., 2014). Regardless, SGNNCs have great potential to reprogram into PAN-like neurons. Previous studies have shown that iNs typically differentiate into neuronal-subtypes consistent with the location of where source cells had been extracted (Liu et al., 2015; Masserdotti et al., 2015). It is unclear what causes this effect. Perhaps cells within the same niche experience the same chemical signals and stresses during development, and are primed toward a specific subtype when reprogramming factors, such as *Ascl1*, are ectopically expressed. Therefore, SGNNCs are likely to reprogram into glutamatergic PAN-like neurons, which have the potential to re-establish a peripheral auditory circuit for future experiments *in vivo*.

The iNs we generated from SGNNCs demonstrated some neuronal characteristics similar to PANs. These iNs were observed to dramatically change their appearance and form bipolar structures with neurite-like extensions that projected toward PAN targets, sensory hair cells and cochlear nucleus neurons. These iNs also positively expressed key neuronal markers such as βIII-tubulin and MAP2. However, when examining the transcriptome of these cells it was clear that some characteristics of SGNNCs persisted in converted cells, at least after the 7-day time point we examined (Figure 5E, 6C). We used PCA (Figure 5B) to analyze factors responsible for the difference between iNs and PANs (Verhoeckx et al., 2004; Hu et al., 2013; Maehara et al., 2015). PC1 accounted for 79% of the variance observed, and the three-groups were arranged in the order VC-iN-PAN. Using our data, it is possible to create a list of genes with positive loading factors based on PC1 and this allows us to uncover the genes that were likely responsible for the principal difference between PANs and iNs. Amongst these genes, 13 were transcription factors (Supplemental Table S1), including *Gata3* (PC1; 0.044, PC2; 0.043). In the future, if we can generate iNs that express some of these genes then we may be able to obtain neurons that are closer to endogenous PANs. This list may be informative in setting criteria to judge the similarity between iNs and PANs for future studies. Since we used P1 spiral ganglia for our studies this may have influenced gene expression. This was an advantageous first step to examine the possibility of reprogramming from SGNNCs to iNs, but future studies will need to analyze adult PANs and iNs from adult SGNNCs as adults are the most likely target for regeneration of PANs.

Previous studies that described the conversion of iNs from fibroblasts, embryonic stem cells (Chanda et al., 2014) or juvenile astrocytes (Liu et al., 2015) analyzed cells after longer periods of culturing than we used, 4 or more weeks vs. 1 week in our study. Although we generated neuron-like cells positive for mature neuronal markers, longer culturing times may have been necessary for iNs to completely change cell identity. However, given that our mode of gene delivery was through Lipofection, instead of viral, and our iNs developed in culture surrounded by mainly glia, instead of other cell types, these differences in transfection and culturing conditions might make it difficult to compare the timelines required for iNs to achieve maturity in our study vs. others. Perhaps cells are more competent to convert into iNs when ectopic gene expression occurs through a preferred method or if cells are co-cultured with others that support neuron growth like glia. Additionally, there are different cell-type-specific prerequisites for reprogramming amongst starter cells (Gascón et al., 2017). Since SGNNCs are a heterogeneous cell population that includes Schwann cells and fibroblasts, different cell types may have been converted to differing degrees. Our RNAseq data suggests that both Schwann cells and fibroblasts had the ability to convert into iNs since our iN groups retained expression of genes from each respective starting cell type (Figure 5E). Hence, it may be worthwhile to discover which cell population has more potential for conversion into neurons that are more PAN-like. Furthermore, it would be interesting to create a developmental portrait of cellular reprogramming, identifying changes from the early stages of a susceptible cell to a completely remodeled one. Single-cell sequencing (Burns et al., 2015; Kwan, 2016) is one method that could offer a high resolution look at the transcriptome during transitions in cellular reprograming (Treutlein et al., 2016). This could be useful for determining the reasons why some cells fail to reprogram, the transcription factors that can help overcome reprogramming barriers and the conditions needed to make iNs more PAN-like; thus, providing insight on how to improve the efficiency of neuronal induction.

It should be noted that the VC groups sequenced in our study were SGNNCs cultured *in vitro* and then transfected with the empty DsRed vector. Mechanical stress during dissociation and culturing conditions may have changed the genomic profile of SGNNCs to appear more similar to iNs than SGNNCs *in situ*. Further experiments should be performed to clarify how primary SGNNCs compare to the VC group. On the other hand, SGNNCs in culture may partially mimic a damaged environment. Since our ultimate goal is to directly reprogram SGNNCs into PANs *in vivo*, where endogenous PANs have degenerated, our experimental paradigm *in vitro* may benefit from an environment where cells are in the process of recovering from injury. Schwann cells proliferate after PAN injury (Lang et al., 2011), creating a generous pool of source cells that will not be depleted after reprogramming. After injury, Schwann cells also upregulate Sox2, a transcription factor that has previously been used to induce neurons from astrocytes and Ng2 glia, and is also important in glial proliferation after injury (Lang et al., 2011; Heinrich et al., 2014).

Our results demonstrate that iNs extend neurites toward peripheral cochlear hair cells and central cochlear nucleus neurons *in vitro*. Future studies should determine whether iNs form connections *in vitro* as further evidence to suggest that iNs *in vivo* will be able to establish a circuit between the peripheral and central auditory systems, an essential step for functional recovery. Neuronal reprogramming *in vivo* faces many challenges for implementation such as the influence of neighboring cells in the damaged environment and the limited optimal time window for direct conversion after injury (See review Gascón et al., 2017). Yet, even with this limitation there have been promising results indicating that neurons reprogrammed *in vivo*, mainly glutamatergic, can integrate into pre-existing circuits (Caiazzo et al., 2011; Kim et al., 2011; Torper et al., 2015). In fact, one study has succeeded in demonstrating functional recovery by converting astrocytes into dopaminergic neurons in a mouse model of Parkinson's disease (Rivetti di Val Cervo et al., 2017). Future study is warranted to clarify what is needed for successful *in vivo* reprogramming and integration for PAN regeneration. Thus, the next goal is to perform cellular reprograming *in viv*o using a preclinical animal model of neuropathy toward the ultimate target of clinical application. The current study provides the first proof-of-principle evidence for endogenous regeneration indicating that SGNNCs can be reprogrammed into iNs *in vitro* for the potential treatment of hearing loss.

## Author contributions

TN, SM, KN, AD: Conceptualization; TN, SM, JN, YA, YO, KN, AD: Formal analysis; TN, JN, YO, KN, AD: Funding acquisition; TN, SM, YA, RU, KN, AD: Investigation; AD, Project administration; AD: Supervision; TN, SM, YA, KN: Validation; TN, SM, RU, KN: Visualization; TN, SM, KN, AD: Writing–original draft; TN, SM, KN, AD: Writing–review and editing.

### Conflict of interest statement

The authors declare that the research was conducted in the absence of any commercial or financial relationships that could be construed as a potential conflict of interest.

## References

[B1] Abbas Y. M. Pichlmair A. Górna M. W. Superti-Furga G. Nagar B. (2013). Structural basis for viral 5'-PPP-RNA recognition by human IFIT proteins. Nature 494, 60–64. 10.1038/nature11783 23334420 PMC4931921

[B2] Ahmed M. Xu J. Xu P.-X. (2012). Eya1 and Six1 drive the neuronal developmental program in cooperation with the SWI/SNF chromatin-remodeling complex and Sox2 in the mammalian inner ear. Development 139, 1965–1977. 10.1242/dev.071670 22513373 PMC3347689

[B3] Ahmed T. Van der Jeugd A. Blum D. Galas M.-C. D'Hooge R. Buee L.. (2014). Cognition and hippocampal synaptic plasticity in mice with a homozygous tau deletion. Neurobiol. Aging 35, 2474–2478. 10.1016/j.neurobiolaging.2014.05.005 24913895

[B4] Appler J. M. Lu C. C. Druckenbrod N. R. Yu W.-M. Koundakjian E. J. Goodrich L. V. (2013). Gata3 is a critical regulator of cochlear wiring. J. Neurosci. 33, 3679–3691. 10.1523/JNEUROSCI.4703-12.2013 23426694 PMC3613247

[B5] Ashburner M. Ball C. A. Blake J. A. Botstein D. Butler H. Cherry J. M.. (2000). Gene ontology: tool for the unification of biology. Nat. Genet. 25, 25–29. 10.1038/75556 10802651 PMC3037419

[B6] Barclay M. Ryan A. F. Housley G. D. (2011). Type I vs type II spiral ganglion neurons exhibit differential survival and neuritogenesis during cochlear development. Neural Dev. 6:33. 10.1186/1749-8104-6-33 21989106 PMC3207869

[B7] Bell D. Streit A. Gorospe I. Varela-Nieto I. Alsina B. Giraldez F. (2008). Spatial and temporal segregation of auditory and vestibular neurons in the otic placode. Dev. Biol. 322, 109–120. 10.1016/j.ydbio.2008.07.011 18674529

[B8] Bermingham-McDonogh O. Oesterle E. C. Stone J. S. Hume C. R. Huynh H. M. Hayashi T. (2006). Expression of Prox1 during mouse cochlear development. J. Comp. Neurol. 496, 172–186. 10.1002/cne.20944 16538679 PMC2572724

[B9] Breuskin I. Bodson M. Thelen N. Thiry M. Borgs L. Nguyen L. (2010). Glial but not neuronal development in the cochleo-vestibular ganglion requires Sox10. J. Neurochem. 114, 1827–1839. 10.1111/j.1471-4159.2010.06897.x 20626560

[B10] Burns J. C. Kelly M. C. Hoa M. Morell R. J. Kelley M. W. (2015). Single-cell RNA-Seq resolves cellular complexity in sensory organs from the neonatal inner ear. Nat. Commun. 6:8557. 10.1038/ncomms9557 26469390 PMC4634134

[B11] Caccamo D. Katsetos C. D. Herman M. M. Frankfurter A. Collins V. P. Rubinstein L. J. (1989). Immunohistochemistry of a spontaneous murine ovarian teratoma with neuroepithelial differentiation. Neuron-associated beta-tubulin as a marker for primitive neuroepithelium. Lab. Invest. 60, 390–398. 2467076

[B12] Caiazzo M. Dell'Anno M. T. Dvoretskova E. Lazarevic D. Taverna S. Leo D.. (2011). Direct generation of functional dopaminergic neurons from mouse and human fibroblasts. Nature 476, 224–227. 10.1038/nature10284 21725324

[B13] Chanda S. Ang C. E. Davila J. Pak C. Mall M. Lee Q. Y.. (2014). Generation of induced neuronal cells by the single reprogramming factor ASCL1. Stem Cell Rep. 3, 282–296. 10.1016/j.stemcr.2014.05.020 25254342 PMC4176533

[B14] Chen W. C. Davis R. L. (2006). Voltage-gated and two-pore-domain potassium channels in murine spiral ganglion neurons. Hear. Res. 222, 89–99. 10.1016/j.heares.2006.09.002 17079103

[B15] Chen W. Jongkamonwiwat N. Abbas L. Eshtan S. J. Johnson S. L. Kuhn S.. (2012). Restoration of auditory evoked responses by human ES-cell-derived otic progenitors. Nature 490, 278–282. 10.1038/nature11415 22972191 PMC3480718

[B16] Chiquet Ehrismann R. Chiquet M. (2003). Tenascins: regulation and putative functions during pathological stress. J. Pathol. 200, 488–499. 10.1002/path.1415 12845616

[B17] Dabdoub A. Fritzsch B. Popper A. N. Fay R. R. (2015). The Primary Auditory Neurons of the Mammalian Cochlea. New York, NY: Springer.

[B18] Dabdoub A. Nishimura K. (2017). Cochlear implants meet regenerative biology: state of the science and future research directions. Otol. Neurotol. 38, e232–e236. 10.1097/MAO.0000000000001407 28806331

[B19] Danielson K. G. Baribault H. Holmes D. F. Graham H. Kadler K. E. Iozzo R. V. (1997). Targeted disruption of decorin leads to abnormal collagen fibril morphology and skin fragility. J. Cell Biol. 136, 729–743. 10.1083/jcb.136.3.729 9024701 PMC2134287

[B20] Diensthuber M. Zecha V. Wagenblast J. Arnhold S. Edge A. S. B. Stöver T. (2014). Spiral ganglion stem cells can be propagated and differentiated into neurons and glia. Biores. Open Access 3, 88–97. 10.1089/biores.2014.0016 24940560 PMC4048968

[B21] Evsen L. Sugahara S. Uchikawa M. Kondoh H. Wu D. K. (2013). Progression of neurogenesis in the inner ear requires inhibition of Sox2 transcription by neurogenin1 and neurod1. J. Neurosci. 33, 3879–3890. 10.1523/JNEUROSCI.4030-12.2013 23447599 PMC3865497

[B22] Flores-Otero J. Davis R. L. (2011). Synaptic proteins are tonotopically graded in postnatal and adult type I and type II spiral ganglion neurons. J. Comp. Neurol. 519, 1455–1475. 10.1002/cne.22576 21452215 PMC3233875

[B23] Gascón S. Masserdotti G. Russo G. L. Götz M. (2017). Direct neuronal reprogramming: achievements, hurdles, and new roads to success. Cell Stem Cell 21, 18–34. 10.1016/j.stem.2017.06.011 28686866

[B24] Guo Z. Zhang L. Wu Z. Chen Y. Wang F. Chen G. (2014). *In vivo* direct reprogramming of reactive glial cells into functional neurons after brain injury and in an Alzheimer's disease model. Cell Stem Cell 14, 188–202. 10.1016/j.stem.2013.12.001 24360883 PMC3967760

[B25] Hafidi A. Fellous A. Ferhat L. Romand M. R. Romand R. (1992). Developmental differentiation of Map2 expression in the central versus the peripheral and efferent projections of the inner ear. J. Comp. Neurol. 323, 423–431. 10.1002/cne.903230309 1281171

[B26] Hallworth R. Ludueña R. F. (2000). Differential expression of β tubulin isotypes in the adult gerbil cochlea. Hear. Res. 148, 161–172. 10.1016/S0378-5955(00)00149-0 10978833

[B27] Heinrich C. Bergami M. Gascón S. Lepier A. Viganò F. Dimou L.. (2014). Sox2-mediated conversion of NG2 glia into induced neurons in the injured adult cerebral cortex. Stem Cell Rep. 3, 1000–1014. 10.1016/j.stemcr.2014.10.007 25458895 PMC4264057

[B28] Heinrich C. Blum R. Gascón S. Masserdotti G. Tripathi P. Sanchez R.. (2010). Directing astroglia from the cerebral cortex into subtype specific functional neurons. PLoS Biol. 8:e1000373. 10.1371/journal.pbio.1000373 20502524 PMC2872647

[B29] Hindson B. J. Ness K. D. Masquelier D. A. Belgrader P. Heredia N. J. Makarewicz A. J.. (2011). High-throughput droplet digital PCR system for absolute quantitation of DNA copy number. Anal. Chem. 83, 8604–8610. 10.1021/ac202028g 22035192 PMC3216358

[B30] Hu J. Locasale J. W. Bielas J. H. O'Sullivan J. Sheahan K. Cantley L. C.. (2013). Heterogeneity of tumor-induced gene expression changes in the human metabolic network. Nat. Biotechnol. 31, 522–529. 10.1038/nbt.2530 23604282 PMC3681899

[B31] Huang D. W. Sherman B. T. Lempicki R. A. (2009). Systematic and integrative analysis of large gene lists using DAVID bioinformatics resources. Nat. Protoc. 4, 44–57. 10.1038/nprot.2008.211 19131956

[B32] Jolliffe I. (1988). Principal Component Analysis 2nd Edn. Chichester: John Wiley & Sons, Ltd.

[B33] Kanehisa M. Goto S. (2000). KEGG: kyoto encyclopedia of genes and genomes. Nucleic Acids Res. 28, 27–30. 10.1093/nar/28.1.27 10592173 PMC102409

[B34] Karalay O. (2011). Prospero-related homeobox 1 gene (Prox1) is regulated by canonical Wnt signaling and has a stage-specific role in adult hippocampal neurogenesis. Proc. Natl. Acad. Sci. U.S.A. 108, 5807–5812. 10.1073/pnas.1013456108 21436036 PMC3078392

[B35] Khalifa S. A. Friberg U. Illing R.-B. Rask-Andersen H. (2003). Synaptophysin immunohistochemistry in the human cochlea. Hear. Res. 185, 35–42. 10.1016/S0378-5955(03)00228-4 14599690

[B36] Kim D. Langmead B. Salzberg S. L. (2015). HISAT: a fast spliced aligner with low memory requirements. Nat. Methods 12, 357–360. 10.1038/nmeth.3317 25751142 PMC4655817

[B37] Kim D. Pertea G. Trapnell C. Pimentel H. Kelley R. Salzberg S. L. (2013). TopHat2: accurate alignment of transcriptomes in the presence of insertions, deletions and gene fusions. Genome Biol. 14:R36. 10.1186/gb-2013-14-4-r36 23618408 PMC4053844

[B38] Kim J. Efe J. A. Zhu S. Talantova M. Yuan X. Wang S.. (2011). Direct reprogramming of mouse fibroblasts to neural progenitors. Proc. Natl. Acad. Sci. U.S.A. 108, 7838–7843. 10.1073/pnas.1103113108 21521790 PMC3093517

[B39] Kim K. K. Adelstein R. S. Kawamoto S. (2009). Identification of neuronal nuclei (NeuN) as Fox-3, a new member of the Fox-1 gene family of splicing factors. J. Biol. Chem. 284, 31052–31061. 10.1074/jbc.M109.052969 19713214 PMC2781505

[B40] Kim W. Y. Fritzsch B. Serls A. Bakel L. A. Huang E. J. Reichardt L. F.. (2001). NeuroD-null mice are deaf due to a severe loss of the inner ear sensory neurons during development. Development 128, 417–426. 11152640 10.1242/dev.128.3.417 PMC2710102

[B41] Kim Y. J. Ibrahim L. A. Wang S. Yuan W. Evgrafov O. V. Knowles J. A.. (2016). EphA7 regulates spiral ganglion innervation of cochlear hair cells. Dev. Neurobiol. 76, 452–469. 10.1002/dneu.22326 26178595 PMC4853368

[B42] Kopan R. Ilagan M. X. G. (2009). The canonical Notch signaling pathway: unfolding the activation mechanism. Cell 137, 216–233. 10.1016/j.cell.2009.03.045 19379690 PMC2827930

[B43] Kujawa S. G. Liberman M. C. (2009). Adding insult to injury: cochlear nerve degeneration after “temporary” noise-induced hearing loss. J. Neurosci. 29, 14077–14085. 10.1523/JNEUROSCI.2845-09.2009 19906956 PMC2812055

[B44] Kwan K. Y. (2016). Single-cell transcriptome analysis of developing and regenerating spiral ganglion neurons. Curr. Pharmacol. Rep. 2, 211–220. 10.1007/s40495-016-0064-z 28758056 PMC5531199

[B45] Lang H. Li M. Kilpatrick L. A. Zhu J. Samuvel D. J. Krug E. L.. (2011). Sox2 up-regulation and glial cell proliferation following degeneration of spiral ganglion neurons in the adult mouse inner ear. J. Assoc. Res. Otolaryngol. 12, 151–171. 10.1007/s10162-010-0244-1 21061038 PMC3046328

[B46] Lang H. Xing Y. Brown L. N. Samuvel D. J. Panganiban C. H. Havens L. T.. (2015). Neural stem/progenitor cell properties of glial cells in the adult mouse auditory nerve. Sci. Rep. 5:13383. 10.1038/srep13383 26307538 PMC4549618

[B47] Liberman M. C. Kujawa S. G. (2017). Cochlear synaptopathy in acquired sensorineural hearing loss: manifestations and mechanisms. Hear. Res. 349, 138–147. 10.1016/j.heares.2017.01.003 28087419 PMC5438769

[B48] Liu M. Pereira F. A. Price S. D. Chu M. J. Shope C. Himes D.. (2000). Essential role of BETA2/NeuroD1 in development of the vestibular and auditory systems. Genes Dev. 14, 2839–2854. 10.1101/gad.840500 11090132 PMC317056

[B49] Liu Y. Miao Q. Yuan J. Han S. Zhang P. Li S.. (2015). Ascl1 converts dorsal midbrain astrocytes into functional neurons *in vivo*. J. Neurosci. 35, 9336–9355. 10.1523/JNEUROSCI.3975-14.2015 26109658 PMC6605193

[B50] Love M. I. Huber W. Anders S. (2014). Moderated estimation of fold change and dispersion for RNA-seq data with DESeq2. Genome Biol. 15:550. 10.1186/s13059-014-0550-8 25516281 PMC4302049

[B51] Lu C. C. Appler J. M. Houseman E. A. Goodrich L. V. (2011). Developmental profiling of spiral ganglion neurons reveals insights into auditory circuit assembly. J. Neurosci. 31, 10903–10918. 10.1523/JNEUROSCI.2358-11.2011 21795542 PMC3167573

[B52] Ma Q. Chen Z. del Barco Barrantes I. de la Pompa J. L. Anderson D. J. (1998). Neurogenin1 is essential for the determination of neuronal precursors for proximal cranial sensory ganglia. Neuron 20, 469–482. 10.1016/S0896-6273(00)80988-5 9539122

[B53] Maehara K. Harada A. Sato Y. Matsumoto M. Nakayama K. I. Kimura H.. (2015). Tissue-specific expression of histone H3 variants diversified after species separation. Epigenetics Chromatin 8:35. 10.1186/s13072-015-0027-3 26388943 PMC4574566

[B54] Masserdotti G. Gillotin S. Sutor B. Drechsel D. Irmler M. Jørgensen H. F.. (2015). Transcriptional mechanisms of proneural factors and REST in regulating neuronal reprogramming of astrocytes. Cell Stem Cell 17, 74–88. 10.1016/j.stem.2015.05.014 26119235 PMC4509553

[B55] Mortazavi A. Williams B. A. McCue K. Schaeffer L. Wold B. (2008). Mapping and quantifying mammalian transcriptomes by RNA-Seq. Nat. Methods 5, 621–628. 10.1038/nmeth.1226 18516045 PMC13303166

[B56] Nayagam B. A. Muniak M. A. Ryugo D. K. (2011). The spiral ganglion: connecting the peripheral and central auditory systems. Hear. Res. 278, 2–20. 10.1016/j.heares.2011.04.003 21530629 PMC3152679

[B57] Nishimura K. Nakagawa T. Sakamoto T. Ito J. (2012). Fates of murine pluripotent stem cell-derived neural progenitors following transplantation into mouse cochleae. Cell Transplant. 21, 763–771. 10.3727/096368911X623907 22305181

[B58] Nishimura K. Noda T. Dabdoub A. (2017). Dynamic expression of Sox2, Gata3, and Prox1 during primary auditory neuron development in the mammalian cochlea. PLoS ONE 12:e0170568. 10.1371/journal.pone.0170568 28118374 PMC5261741

[B59] Nishimura K. Weichert R. M. Liu W. Davis R. L. Dabdoub A. (2014). Generation of induced neurons by direct reprogramming in the mammalian cochlea. Neuroscience 275, 125–135. 10.1016/j.neuroscience.2014.05.067 24928351

[B60] Ohtsuka M. Inoko H. Kulski J. K. Yoshimura S. (2008). Major histocompatibility complex (Mhc) class Ib gene duplications, organization and expression patterns in mouse strain C57BL/6. BMC Genomics 9:178. 10.1186/1471-2164-9-178 18416856 PMC2375909

[B61] Oshima K. Teo D. T. W. Senn P. Starlinger V. Heller S. (2007). LIF promotes neurogenesis and maintains neural precursors in cell populations derived from spiral ganglion stem cells. BMC Dev. Biol. 7:112. 10.1186/1471-213X-7-112 17935626 PMC2080640

[B62] Pang Z. P. Yang N. Vierbuchen T. Ostermeier A. Fuentes D. R. Yang T. Q.. (2011). Induction of human neuronal cells by defined transcription factors. Nature 476, 220–223. 10.1038/nature10202 21617644 PMC3159048

[B63] Pertea M. Kim D. Pertea G. M. Leek J. T. Salzberg S. L. (2016). Transcript-level expression analysis of RNA-seq experiments with HISAT, stringtie and ballgown. Nat. Protoc. 11, 1650–1667. 10.1038/nprot.2016.095 27560171 PMC5032908

[B64] Provenzano M. J. Minner S. A. Zander K. Clark J. J. Kane C. J. Green S. H. (2011). p75NTR expression and nuclear localization of p75NTR intracellular domain in spiral ganglion Schwann cells following deafness correlate with cell proliferation. Mol. Cell. Neurosci. 47, 306–315. 10.1016/j.mcn.2011.05.010 21658451 PMC3137691

[B65] Puligilla C. Dabdoub A. Brenowitz S. D. Kelley M. W. (2010). Sox2 induces neuronal formation in the developing mammalian cochlea. J. Neurosci. 30, 714–722. 10.1523/JNEUROSCI.3852-09.2010 20071536 PMC2835399

[B66] Radde-Gallwitz K. Pan L. Gan L. Lin X. Segil N. Chen P. (2004). Expression of Islet1 marks the sensory and neuronal lineages in the mammalian inner ear. J. Comp. Neurol. 477, 412–421. 10.1002/cne.20257 15329890 PMC4158841

[B67] Rivetti di Val Cervo P. Romanov R. A. Spigolon G. Masini D. Martín-Montañez E. Toledo E. M.. (2017). Induction of functional dopamine neurons from human astrocytes *in vitro* and mouse astrocytes in a Parkinson's disease model. Nat. Biotechnol. 35, 444–452. 10.1038/nbt.3835 28398344

[B68] Sagers J. E. Landegger L. D. Worthington S. Nadol J. B. Stankovic K. M. (2017). Human cochlear histopathology reflects clinical signatures of primary neural degeneration. Sci. Rep. 7:4884. 10.1038/s41598-017-04899-9 28687782 PMC5501826

[B69] Scarfone E. Dememes D. Sans A. (1991). Synapsin, I., and Synaptophysin expression during ontogenesis of the mouse peripheral vestibular system. J. Neurosci. 11, 1173–1181. 1902873 10.1523/JNEUROSCI.11-05-01173.1991 PMC6575312

[B70] Schmid D. Zeis T. Schaeren-Wiemers N. (2014). Transcriptional regulation induced by cAMP elevation in mouse Schwann cells. ASN Neuro 6, 137–157. 10.1042/AN20130031 24641305 PMC4834722

[B71] Shibata S. B. Cortez S. R. Beyer L. A. Wiler J. A. Di Polo A. Pfingst B. E.. (2010). Transgenic BDNF induces nerve fiber regrowth into the auditory epithelium in deaf cochleae. Exp. Neurol. 223, 464–472. 10.1016/j.expneurol.2010.01.011 20109446 PMC2864331

[B72] Srivastava D. DeWitt N. (2016). *In vivo* cellular reprogramming: the next generation. Cell 166, 1386–1396. 10.1016/j.cell.2016.08.055 27610565 PMC6234007

[B73] Suzuki J. Corfas G. Liberman M. C. (2016). Round-window delivery of neurotrophin 3 regenerates cochlear synapses after acoustic overexposure. Sci. Rep. 6:24907. 10.1038/srep24907 27108594 PMC4842978

[B74] Torper O. Ottosson D. R. Pereira M. Lau S. Cardoso T. Grealish S.. (2015). *In vivo* reprogramming of striatal NG2 glia into functional neurons that integrate into local host circuitry. Cell Rep. 12, 474–481. 10.1016/j.celrep.2015.06.040 26166567 PMC4521079

[B75] Treutlein B. Lee Q. Y. Camp J. G. Mall M. Koh W. Shariati S. A. M.. (2016). Dissecting direct reprogramming from fibroblast to neuron using single-cell RNA-seq. Nature 534, 391–395. 10.1038/nature18323 27281220 PMC4928860

[B76] Tucker K. L. Meyer M. Barde Y. A. (2001). Neurotrophins are required for nerve growth during development. Nat. Neurosci. 4, 29–37. 10.1038/82868 11135642

[B77] Verhoeckx K. C. Bijlsma S. de Groene E. M. Witkamp R. F. van der Greef J. Rodenburg R. J. (2004). A combination of proteomics, principal component analysis and transcriptomics is a powerful tool for the identification of biomarkers for macrophage maturation in the U937 cell line. Proteomics 4, 1014–1028. 10.1002/pmic.200300669 15048983

[B78] Wagner G. P. Kin K. Lynch V. J. (2012). Measurement of mRNA abundance using RNA-seq data: RPKM measure is inconsistent among samples. Theory Biosci. 131, 281–285. 10.1007/s12064-012-0162-3 22872506

[B79] Wang P. Zhang P. Huang J. Li M. Chen X. (2013). Trichostatin A protects against cisplatin-induced ototoxicity by regulating expression of genes related to apoptosis and synaptic function. Neurotoxicology 37, 51–62. 10.1016/j.neuro.2013.03.007 23558232

[B80] Wapinski O. L. Vierbuchen T. Qu K. Lee Q. Y. Chanda S. Fuentes D. R.. (2013). Hierarchical mechanisms for direct reprogramming of fibroblasts to neurons. Cell 155, 621–635. 10.1016/j.cell.2013.09.028 24243019 PMC3871197

[B81] White J. A. Burgess B. J. Hall R. D. Nadol J. B. (2000). Pattern of degeneration of the spiral ganglion cell and its processes in the C57BL/6J mouse. Hear. Res. 141, 12–18. 10.1016/S0378-5955(99)00204-X 10713491

[B82] Whitlon D. S. Tieu D. Grover M. (2010). Purification and transfection of cochlear Schwann cells. J. Neurosci. 171, 23–30. 10.1016/j.neuroscience.2010.08.069 20837108

[B83] Whitlon D. S. Tieu D. Grover M. Reilly B. Coulson M. T. (2009). Spontaneous association of glial cells with regrowing neurites in mixed cultures of dissociated spiral ganglia. J. Neurosci. 161, 227–235. 10.1016/j.neuroscience.2009.03.044 19324078 PMC2855887

[B84] Wise A. K. Tu T. Atkinson P. J. Flynn B. O. Sgro B. E. Hume C.. (2011). The effect of deafness duration on neurotrophin gene therapy for spiral ganglion neuron protection. Hear. Res. 278, 69–76. 10.1016/j.heares.2011.04.010 21557994 PMC3152700

[B85] Wu J. Liu B. Fan J. Zhu Q. Wu J. (2011). Study of protective effect on rat cochlear spiral ganglion after blast exposure by adenovirus-mediated human β-nerve growth factor gene. Am. J. Otolaryngol. 32, 8–12. 10.1016/j.amjoto.2009.08.012 20022668

[B86] Yu W.-M. Appler J. M. Kim Y.-H. Nishitani A. M. Holt J. R. Goodrich L. V. (2013). A Gata3–Mafb transcriptional network directs post-synaptic differentiation in synapses specialized for hearing. Elife 2:e01341. 10.7554/eLife.01341 24327562 PMC3851837

[B87] Yuan Y. Shi F. Yin Y. Tong M. Lang H. Polley D. B.. (2014). Ouabain-induced cochlear nerve degeneration: synaptic loss and plasticity in a mouse model of auditory neuropathy. J. Assoc. Res. Otolaryngol. 15, 31–43. 10.1007/s10162-013-0419-7 24113829 PMC3901858

[B88] Zhou J. Nannapaneni N. Shore S. (2007). Vessicular glutamate transporters 1 and 2 are differentially associated with auditory nerve and spinal trigeminal inputs to the cochlear nucleus. J. Comp. Neurol. 500, 777–787. 10.1002/cne.21208 17154258

[B89] Zhou Q. Anderson D. J. (2002). The bHLH transcription factors Olig2 and Olig1 couple neuronal and glial subtype specification. Cell 109, 61–73. 10.1016/S0092-8674(02)00677-3 11955447

