# Supplementary material for: Direct Reprogramming of Spiral Ganglion Non-neuronal Cells into Neurons: Toward Ameliorating Sensorineural Hearing Loss by Gene Therapy
Source: Front Cell Dev Biol. 2018 Feb 14;6:16. doi: 10.3389/fcell.2018.00016 (PMC5817057; doi:10.3389/fcell.2018.00016)
Supplement: Supplementary file 1 [file Table1.DOCX]

Supplementary Material

Direct reprogramming of spiral ganglion non-neuronal cells into neurons: Towards ameliorating sensorineural hearing loss by gene therapy

Teppei Noda, Steven J. Meas, Jumpei Nogami, Yutaka Amemiya, Ryutaro Uchi, Yasuyuki Ohkawa, Koji Nishimura, Alain Dabdoub

*** Correspondence:** Dr. Alain Dabdoub: Alain.Dabdoub@sri.utoronto.ca

**Supplemental Figure S1. A heatmap of unsupervised hierarchical clustering.** Unsupervised hierarchical clustering of the 17,601 genes detected among endogenous neurons (PAN), induced neurons (iN), and control (VC) groups. High TPM values are indicated in red and low TPM values are indicated in blue. Samples clustered well within each group. The iN group contained a mix of genes expressed in both PAN and VC groups.

**Supplemental Video:** Time-lapse video microscopy of SGNNCs from P1 Tau-EGFP mice transfected with *Ascl1-DsRed* was performed with Vivaview (Olympus) at a constant temperature of 37° C and 5% CO_2_. Phase contrast image overlayed with DsRed fluorescence (left) and Tau-EGFP fluorescence (right). Images were acquired every 30 min for 18 h starting 4 days after transfection. The transfected cell changed its shape (left and right image) towards a neuron-like morphology while upregulating Tau-EGFP expression (right image).

**Supplemental Table 1.** Transcription factors with positive loadings both in PC1 and PC2 axes.

| Gene | PC1 | PC2 | PAN (meanTPM) | iN (meanTPM) | VC (meanTPM) |
| --- | --- | --- | --- | --- | --- |
| *Esrrg* | 0.060 | 0.022 | 197.813 | 0.467 | 0.017 |
| *Lin28b* | 0.054 | 0.006 | 12.357 | 0.137 | 0.000 |
| *Ern2* | 0.050 | 0.035 | 3.623 | 0.000 | 0.000 |
| *Fgf23* | 0.049 | 0.017 | 7.893 | 0.050 | 0.000 |
| *Nos1* | 0.047 | 0.017 | 8.087 | 0.083 | 0.013 |
| *Zbtb8b* | 0.046 | 0.001 | 9.993 | 0.463 | 0.027 |
| *Pou6f2* | 0.046 | 0.021 | 6.427 | 0.040 | 0.000 |
| *Tunar* | 0.046 | 0.022 | 2.240 | 0.000 | 0.000 |
| *Isl2* | 0.045 | 0.012 | 32.767 | 0.620 | 0.033 |
| *Ripply2* | 0.044 | 0.021 | 10.573 | 0.000 | 0.000 |
| *Gata3* | 0.044 | 0.043 | 104.467 | 0.503 | 0.480 |
| *Pgr* | 0.043 | 0.028 | 3.063 | 0.023 | 0.040 |
| *Mstn* | 0.035 | 0.067 | 12.043 | 0.010 | 0.347 |
