# Supplementary figures and images for: Direct Reprogramming of Spiral Ganglion Non-neuronal Cells into Neurons: Toward Ameliorating Sensorineural Hearing Loss by Gene Therapy
Source: Front Cell Dev Biol. 2018 Feb 14;6:16. doi: 10.3389/fcell.2018.00016 (PMC5817057; doi:10.3389/fcell.2018.00016)

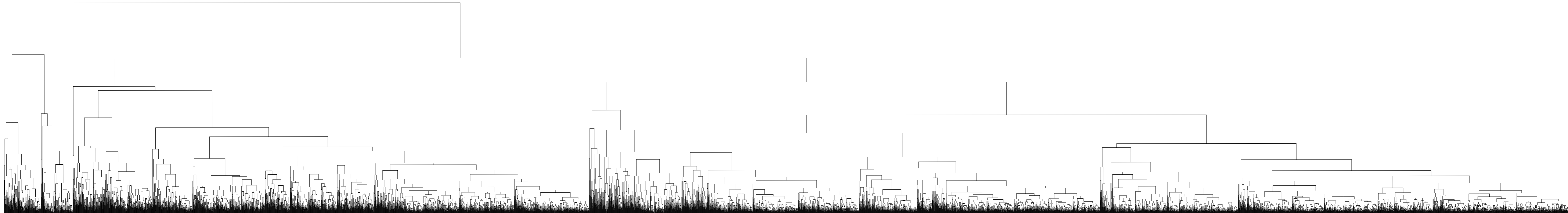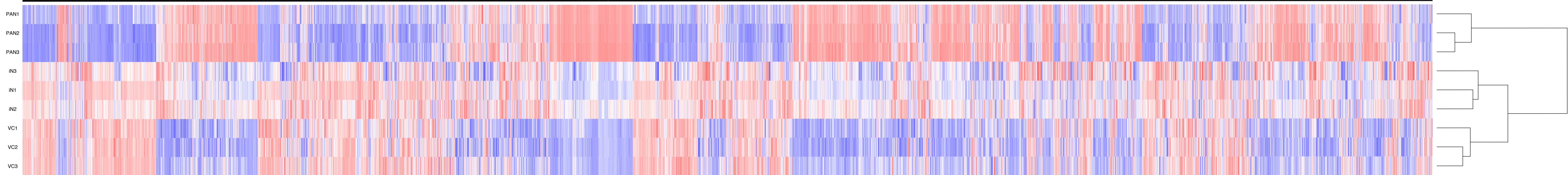

Supplement: Supplementary file 3 [file Image1.PDF]
